# Skin Cancer Detection Based on Extreme Learning Machine and a Developed Version of Thermal Exchange Optimization

**DOI:** 10.1155/2021/9528664

**Published:** 2021-11-03

**Authors:** Shi Wang, Melika Hamian

**Affiliations:** ^1^Department of Computer Engineering, Dongguan Polytechnic, Dongguan 523808, Guangdong, China; ^2^Department of Engineering, Payame Noor University (PNU), Tehran, Iran

## Abstract

Melanoma is defined as a disease that has been incurable in advanced stages, which shows the vital importance of timely diagnosis and treatment. To diagnose this type of cancer early, various methods and equipment have been used, almost all of which required a visit to the doctor and were not available to the public. In this study, an automated and accurate process to differentiate between benign skin pigmented lesions and malignant melanoma is presented, so that it can be used by the general public, and it does not require special equipment and special conditions in imaging. In this study, after preprocessing of the input images, the region of interest is segmented based on the Otsu method. Then, a new feature extraction is implemented on the segmented image to mine the beneficial characteristics. The process is then finalized by using an optimized Deep Believe Network (DBN) for categorization into 2 classes of normal and melanoma cases. The optimization process in DBN has been performed by a developed version of the newly introduced Thermal Exchange Optimization (dTEO) algorithm to obtain higher efficacy in different terms. To show the method's superiority, its performance is compared with 7 different techniques from the literature.

## 1. Introduction

The skin cancer has been known as one of the deadliest types of cancers which has recently witnessed an exponential growth worldwide. With all these interpretations, it can be cured if it is detected in the early stages, and, in most cases, a simple biopsy can prevent cancer from growing [1]. This cancer has been growing dramatically during the recent years and the importance of the initial therapy has been more to be seen. The melanoma skin cancer has been ranked as the 3rd most common malignant cancer among the skin cancer cases [2]. This cancer alters the skin color as a result of the irregular effects of the cells [3]. Notwithstanding this danger, it is known as a curable cancer if it is detected in the early stages. With all these interpretations, early detection of the melanoma from the other types of the benign skin moles is a thought-provoking mission. Any variations in the size, color, or the shape of a skin mole can be considered as initial signs for this cancer.

Based on the statistical information for 2019, skin cancer with 15000 cases is ranked as the fourth most common cancer, and with 1900 deaths cases are ranked as one of the 10th common cancers in the world [4]. The most common method for melanoma detection is by the physicians and their sampling and testing. However, in some cases, even experienced specialists make some mistakes in melanoma detection. Furthermore, laboratory sampling and testing is a time-consuming and expensive experiment that bothers the patients. Therefore, designing a (computer) system that can detect malignant lesions will be very useful. According to researches, the effectiveness of the computer-aided system is more accurate than that of a specialist doctor. Recently, many research works are accomplished for automatic early detection of skin cancers. Also, the high capability of the medical imaging assists the medical doctors to lessen the diagnosis difficulty of this work and to enhance the speed of the initial diagnosis of the disease.

The application of Artificial Neural Networks (ANNs) in different parts of image processing and machine vision has been increasing. The ANNs are imitated from the human brain actions and are used in a variety of applications, from medicine to economics and engineering [5].

The ANN is made up of lots of highly unified processing components called neurons which can solve problems with their interconnectedness. Artificial Neural Networks, like humans, learn by example to solve different kinds of problems from pattern recognition to information classification throughout a learning procedure.

Deep learning is a sort of ANNs which uses mathematical techniques to give a structure like the human brain. On the other hand, advances in technology have led to algorithms for optimizing normal neural networks so that we can count the number of neural layers. Neural networks are increased from multiple layers to thousands of layers and thousands of neurons in each layer, which until a few years ago could not have created such a structure [6]. This type of neural network is called a deep learning network. Several methodologies of using different kinds of ANNs and other machine learning techniques were introduced for skin cancer diagnosis [7, 8]. For instance, Li et al. [9] proposed a new data synthesis methodology to combine individual skin lesions images and heavily expand them to create noteworthy amounts of data. The study worked on a convolutional neural network (CNN) to provide superior efficiency to the old-style detection and tracking methods. Moreover, the system was trained by humans with simple criteria.

Esteva et al. [10] presented a supervised technique for skin lesions using deep learning. The method's efficiency was verified by twenty-one medical images with two critical binary classifications: malignant and benign groups. Two cases were analyzed here. The first one recognizes the most common cancers, and the second case signifies the deadliest skin cancer identification. The final results indicated the high performance of the proposed method.

Jafari et al. [11] proposed a deep learning-based procedure to provide a precise skin cancer area segmentation. After image denoising and preprocessing, a CNN was utilized for segmentation. They combined local and global structure information and outputted a label for the pixels to create a segmentation mask that indicates the lesion area. This mask was then improved with some postprocessing operators. The empirical results indicated the superiority of the presented method compared to the other techniques.

Mohamed et al. [12] proposed a learning technique to categorize skin lesions to determine melanoma. The method was based on applying a CNN with multiple outlines. The results of the suggested technique were performed by 14 layers into the International Skin Imaging Collaboration (ISIC) dataset. Final results indicated successful ratio for the method in the segmentation of the skin cancer area.

Xu et al. [2] proposed an automatic diagnosis method for melanoma. The presented technique was based on an optimized image segmentation methodology along with a CNN optimized by satin bowerbird optimization (SBO). After segmentation, feature extraction was implemented on the processed image. The main features were then selected using the SBO algorithm to trim additional information. At last, the results were presented by performing an SVM into two groups of normal and melanoma cases. The method's results were authenticated by comparison with other techniques from the literature to indicate its performance.

**Figure 1:**
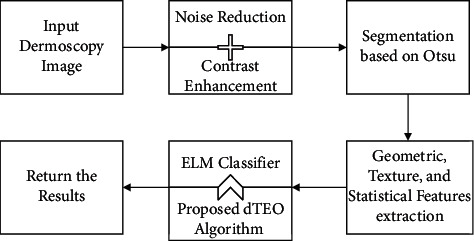
The overall procedure of the suggested method.

As the research background shows, the application of deep learning, including CNNs in medical images processing, especially in skin lesion diagnosis, has been exponentially increasing. The present study proposes an optimized technique of skin lesion segmentation using a new design of the Thermal Exchange Optimization algorithm which has been used to reach this purpose with higher performance. The overall technique for the proposed technique has been shown in Figure 1.

Therefore, the main contributions of the present study can be highlighted as follows:

- A new computer-aided method for the diagnosis of malignant melanoma
- Segmenting the region of interest based on the Otsu method
- Feature extraction to extract the beneficial features
- A new optimized Deep Believe Network (DBN) to classify the melanoma cases
- Optimization based on a developed version of the newly introduced Thermal Exchange Optimization (dTEO) algorithm

## 2. Skin Cancer Preprocessing

Medical imaging is the method and process used to create images of the human body to examine or advance medical science and is widely used in medicine [13]. Diagnostic images are the subject of many scans, examinations, and images used in medicine. Researches have shown that medical images taken are highly susceptible to vulnerability to noise and other factors such as low contrast. Here are two simple ways to do this purpose.

### 2.1. Noise Reduction

Images noise removal is a significant part of all medical image processing. This process tries to reduce or remove the noise from the original image. Features that expose them to noise are available on all recording devices [14]. The noise may be white or random noise. Noise reduction recreates and restores the noisy image to its original model based on ideal models in which we analyze how this method is done. The noise has a major impact on the image in certain situations, particularly through the identification of the image edge that needs differentiation which increases the influence of high-frequency pixels that specifically contain noise [15, 16].

The noise in medical images is typically Gaussian noise. Gaussian noise is dispersed in the image and is located between the image main pixels, which makes some inconsistencies for the next operations, and because the processing must be done only on the image main pixels, it requires a proper noise elimination for this purpose. Noise pixels are usually recognized as a different pixel among a group of pixels in the original image that differs from adjacent pixels in different terms such as light intensity and transparency. Wang-Mendel algorithm is an efficient fuzzy-based noise removal tool [17]. Because of the simple conception of the fuzzy techniques, they are popular for different applications. Furthermore, because of their speediness as an early-stage process, they are very useful for producing the initial fuzzy model [18]. In this method, the input and the output datasets show the behavior of the solved state of the problem. The production of the rule database is performed as follows:

1. Consider fuzzy isolation from the space that includes the input variables, which is achieved by the knowledge of an expert along with normalization. Then, it splits it into equivalent or unequal sections by doing a fuzzy-based method for parting the space of input variable. Then a form of the membership function is chosen and each component is given as a fuzzy package. Afterward, the membership and fuzzy set has been established to all the parts.
2. A set of candidate language rules are produced, which are generated by choosing the most inclusive rules for the samples.
3. Validity degree is assigned for the laws, which is achieved by multiplying the membership function values for all the components.
4. The final rules database is obtained from the rules of the candidate language set, which is done by categorizing these rules into some groups, where the groups include the rules of candidates with the same assumption. The final rule is obtained by selecting the highest degree for all the groups.

### 2.2. Image Contrast Enhancement

**Figure 2:**
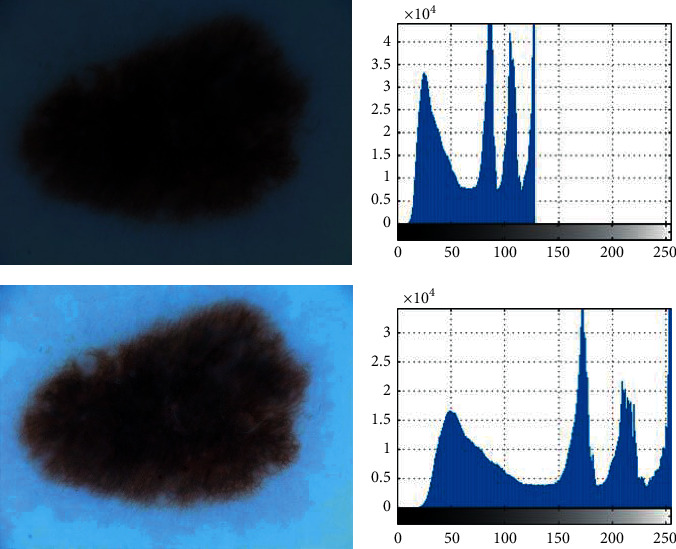
Two examples of the preprocessing of the image: (a) original image; (b) PDF of (a).

Dermoscopy images typically lack high image contrast, which makes them impossible to process next stages. The concern arises from multiple circumstances, such as the poor quality of measuring instruments and cameras, the low degree of the user interface in photography, the environmental factors, and the prevalence of noise. Sometimes, any crucial details from the images disappear from the above examples, rendering the processing too complex [19]. Contrast enhancement is a method for addressing the problems of contrast consistency. In this research, image contrast enhancement is used to enhance and help demonstrate the specifics of the cancer regions. Global enhancement of contrast using the lookup table is used in the present research. For categorizing and storing the received images on the disk, an 8-bit lookup table is used. The strategy is usually developed as follows [20]:

(1) $$\begin{array}{c} {\text{PDF}}_{\text{out}}=\frac{{\text{PDF}}_{\text{In}}-{\text{PDF}}_{\min}}{{\text{PDF}}_{\text{Max}}-{\text{PDF}}_{\min}}, \end{array}$$

where PDF_In_ and PDF_out_ represent the probability density function of the input and the output corrected images, respectively, and PDF_min_ and PDF_Max_ represent the minimum and the maximum probability density levels, respectively.  Figure 2 shows an example of the preprocessed image for more clarification.

## 3. Skin Cancer Segmentation

### 3.1. Color Space

The RGB is the most common and basic color space in images processing, which usually is used for displaying images. Due to the dependence of these three colors, which are considered as base colors in the RGB color space, as well as their dependence on the intensity of ambient light, this color space is less used for main processing. To compensate for this shortcoming, the XYZ color model has been employed. This color space is a quantifiable link between the physiologically supposed colors in human color vision and wavelengths distributions in the electromagnetic visible spectrum . In this color space, the values of *X* and *Z* give color information, and *Y* defines the luminance. To convert color space from RGB to XYZ, the following equation has been used [21]:

(2) $$\begin{array}{c} \left[\begin{array}{c} X\\ Y\\ Z \end{array}\right]=\frac{1}{0.17697}\times \left[\begin{array}{ccc} 0.49 & 0.31 & 0.2\\ 0.17697 & 0.8124 & 0.01063\\ 0 & 0.01 & 0.99 \end{array}\right]\times \left[\begin{array}{c} R\\ G\\ B \end{array}\right]. \end{array}$$

The most significant benefit of *XYZ* color model is that it is completely independent of the device.

### 3.2. Method of Segmentation

In dermoscopy images, the red color space is the only dimension in RGB color space that gives nearly the strength of the images. As mentioned before, both *X* and *Z* values give related information from the color for the XYZ color space. Therefore, the red (R) dimension and the *X* dimension are just normalized for the segmentation; that is [21],

(3) $$\begin{array}{c} \hat{R}=\frac{R}{\sqrt{R^{2}+G^{2}+B^{2}}},\\ \hat{X}=\frac{X}{\sqrt{X^{2}+Y^{2}+Z^{2}}}. \end{array}$$

On any pixel of the input images, these normalization values are performed. After this normalization, the Otsu thresholding is used for giving a low-cost segmentation in terms of time complexity.

The Otsu technique is a popular method for proper thresholding of the images. The method is based on the conception of the variance of the intergroup maximization and the intragroup minimizing of the pixels. The global threshold has a problem when the image background resolution is inadequate. A local threshold can be used to eliminate the heterogeneity effect. This problem is solved by image preprocessing to eliminate inhomogeneities and apply a global threshold to the processed image. Based on Otsu technique, threshold level is comprehensively searched to provide minimum in-between variance class, which is expressed as follows:

(4) $$\begin{array}{c} v\left(t\right)=\omega_{1}\left(t\right)v_{1}^{2}\left(t\right)+\omega_{2}\left(t\right)v_{2}^{2}\left(t\right), \end{array}$$

where *ω*_*i*_ denotes the probability of two separate classes with a threshold value of *t*. *σ*_*i*_^2^ in this case is the value of the variance of these classes. In fact, Otsu shows that minimizing the amount of variance in a class is like maximizing interclass variance:

(5) $$\begin{array}{c} v_{b}^{2}\left(t\right)=v^{2}-v_{\omega }^{2}\left(t\right)=\omega_{1}\left(t\right)\omega_{2}\left(t\right){\left[m_{1}\left(t\right)-m_{2}\left(t\right)\right]}^{2}, \end{array}$$

where *v*_*i*_^2^ describes the variance and *m*_*i*_ signifies the mean value that can be updated intermittently. This technique is briefly given as follows:

- Evaluate image histogram and its intensity level probability
- Give initial values of *ω*_*i*_(0) and *v*_*i*_(0) for all levels of possible threshold (*t* = 1, 2,… )
- Renew *ω*_*i*_ and *m*_*i*_
- Calculate *v*_*b*_^2^(*t*)

The optimal threshold here is the maximum of *σ*_*b*_^2^(*t*).

Then, a morphology operation including closing, opening, and filling is used for better results. In this stage, by applying the mathematical filling operator, the extra image holes are filled. This operator is as follows [22]:

(6) $$\begin{array}{c} X_{k}=\left(X_{k-1}⊕D\right)\cap C^{c},\;k=1,2,3\ldots , \end{array}$$

where *A* and *B* represent the area and the constructing component, respectively.

Then, the opening operation has been established on the filled image for eliminating the ignitor particulars with no changes on other gray surfaces. This operator is as follows:

(7) $$\begin{array}{c} C\circ D=\left(C⊖\;D\right)⊕D. \end{array}$$

Finally, for connecting the narrow parts, the closing operation is performed based on the following equation:

(8) $$\begin{array}{c} C\cdot D=\left(C⊕D\right)⊖\;D. \end{array}$$

Here, the structural element in this study is set as a 5 × 5 identity matrix.  Algorithm 1 shows the pseudocode of the image segmentation.

**Figure 3:**
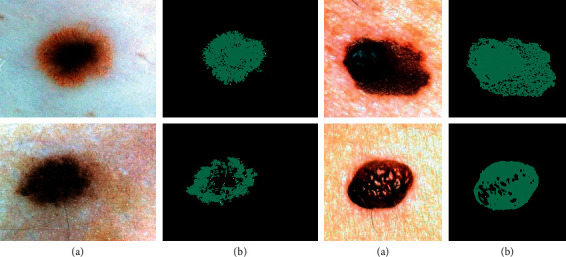
Some samples of the skin cancer segmentation using the suggested technique: (a) the input image and (b) the segmented image.

Figure 3 displays some samples of image segmentation using the suggested segmentation.

## 4. Features Extraction

Feature extraction is the process of collecting more detailed information from the image. The features extraction in this part of processing is utilized for extracting the main features of the segmented skin cancer area to simplify the diagnosis successful. There are numerous features for feature extraction. This research uses statistical features and texture features, and geometric features have been adopted for this purpose.  Table 1 gives the features utilized here.

In the above table, MN describes the size of image, *B*_*p*_ describes the length of external side in the pixel boundary, *p*(*i*, *j*) represents the intensity values of the pixels at position, *μ* defines the mean value, *σ* describes the standard deviation, and *a* and *b* define the major axis and the minor axis, respectively. However, some achieved features are not useful for the process and just increase the method's complexity. To prevent this issue, a feature selection procedure has been utilized. To do so, in this study, an optimized technique has been used. For giving optimal results of features selection, the following cost function has been considered:

(9) $$\begin{array}{c} \text{fitness}=\frac{\left(T_{P}\times T_{N}\right)-\left(F_{P}\times F_{N}\right)}{\sqrt{\left(\left(T_{N}+F_{P}\right)\times \left(T_{P}+F_{P}\right)\times \left(T_{P}+F_{N}\right)\times \left(T_{N}+F_{N}\right)\right)}}, \end{array}$$

where *F*_*P*_ describes the false positive, *F*_*N*_ describes the false negative, *T*_*P*_ defines true positive, and *T*_*N*_ defines the true negative. The idea is to minimize the above equation based on a developed optimization method.

## 5. Extreme Learning Machine (ELM)

**Figure 4:**
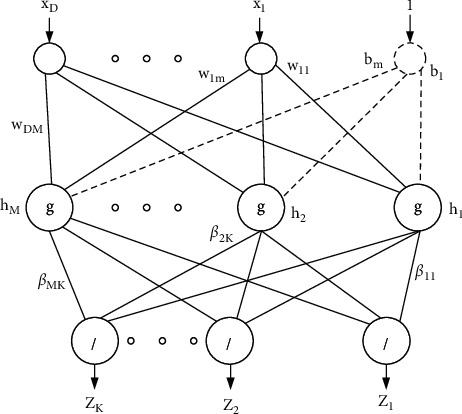
A sample configuration of the ELM network.

One of the neural network models which has recently received many considerations is the ELM model. Some of the advantages for ELM are their high speed in learning, their simple using, and their ability to be used in numerous activation functions and nonlinear kernel functions. The ELM model can provide an integrated template with a wide variety of feature transmissions that can be used in the hidden layer, which can be utilized directly for regression and categorization. This algorithm is a learning method for Single Hidden Neural Networks (SHNN) based on initializing the input biases and weights randomly and the output weights evaluation. Thus, the network can be trained in just a few steps.  Figure 4 shows the arrangement of a simple ELM network.

For a classification problem with *D* dimension and *N* number of training samples [23],

(10) $$\begin{array}{c} \left(x^{\left(n\right)},t^{\left(n\right)}\right),\;\;n=1:N, \end{array}$$

where *x*^(*n*)^ ∈ *ℝ*^*D*^ and *t*^(*n*)^ ∈ *ℝ*^*K*^.

An ELM-based feed-forward neural network can be formulated as follows:

(11) $$\begin{array}{c} \overset{M}{\underset{m=1}{\sum }}\beta_{m}g\left(w_{m}^{T}x^{\left(n\right)}+b_{m}\right)=t^{\left(n\right)}, \end{array}$$

where *g*(·) describes the activation function, *b*_*m*_ describes the bias of the *m*^th^ hidden neuron, *M* signifies the hidden neurons, *w*_*m*_=[*w*_*m*_1__, *w*_*m*_2__,…, *w*_mD_] determines the input weight vector which connects the input neurons to the *m*^th^ the neuron of the hidden layer, and *β*_*m*_=[*β*_*m*_1__, *β*_*m*_2__,…, *β*_mK_] determines the vector of weight that connects the *m*^th^ neuron.

This conception is given as follows:

(12) $$\begin{array}{c} H\beta =T, \end{array}$$

where

(13) $$\begin{array}{c} H={\left[\begin{array}{ccc} g\left(w_{m}^{T}x^{\left(1\right)}+b_{1}\right) & \cdots & g\left(w_{M}^{T}x^{\left(1\right)}+b_{1}\right)\\ \vdots & \ddots & \vdots \\ g\left(w_{1}^{T}x^{\left(N\right)}+b_{1}\right) & \ldots & g\left(w_{M}^{T}x^{\left(N\right)}+b_{M}\right) \end{array}\right]}_{N\times M},\\ H={\left[\beta_{1}^{T},\beta_{2}^{T},\ldots ,\beta_{M}^{T}\right]}_{M\times N}^{T},\\ T={\left[t_{1}^{T},t_{2}^{T},\ldots ,t_{M}^{T}\right]}_{N\times K}^{T} \end{array}$$

Meanwhile, *H* defines a nonsquare matrix. The training samples quantity is bigger than the hidden neurons quantity. Therefore, to solve this problem, the following formulation has been used:

(14) $$\begin{array}{c} \hat{\beta }=H^{†}T, \end{array}$$

where *H*^†^ describes the generalized Moore-Penrose matrix inverse.

Thus, the ELM networks can be shortened to three main steps:

1. Initializing the input weights and bias with random values
2. Evaluation of the hidden neurons' outputs matrix *H*
3. Evaluation of the hidden neurons' outputs weights matrix by equation (16)

## 6. The Developed Thermal Exchange Optimization Algorithm

Optimization is the process of using specific techniques to solve the optimization problems. However, in recent years, the performance of the classic optimization methods due to increasing the complexity and nonlinearity of them has been decreased or even failed [24]. Recently, several methods have been introduced for resolving these problems [25]. Metaheuristics are one class of these methods that have been popular due to their simple and suitable results in various problems. The metaheuristic algorithms are derived from various phenomena from nature, human society, animal hunting behavior, and so forth. Several algorithms have been proposed in this field [26–29], for example, World Cup Optimizer [30], Ant Lion Optimizer (ALO) [31], Chimp Optimization Algorithm [32], Harris Hawks Optimization [33], and mayfly optimization algorithm [34]. In the present research, a novel modified model of the Thermal Exchange Optimization (TEO) algorithm has been presented to achieve optimal results for the considered methodology [5]. The TEO algorithm is a novel metaheuristic technique that is inspired by the temperature of the objects and their position that is switched between cold and warm places, indicating the updated positions. In the following, more explanation about this algorithm has been provided.

### 6.1. The Newton Cooling Law

The tests indicated that the rate of cooling was roughly related to the temperature difference between the environment and the heated object. This is formulated as in the following equation:

(15) $$\begin{array}{c} \frac{\text{d}Q}{\text{d}t}=\alpha \times A\times \left(T_{s}-T_{b}\right), \end{array}$$

where *A* describes the area of the body surface that transmits heat, *Q* signifies the heat, *α* defines the coefficient of heat transfer that is reliant on different cases like object geometry, heat transfer mode, and surface state, and *T*_*b*_ and *T*_*s*_ represent the temperature of the body and the ambient temperature, respectively.

The heat loss in time *dt* is *α* × *A* × (*T*_*s*_ − *T*) d*t*, which states the variation in kept heat as the temperature drops *dT*; in other words,

(16) $$\begin{array}{c} V\times d\times c\times dT=-\alpha \times A\times \left(T-T_{b}\right)\text{ d}t, \end{array}$$

where *d* signifies the density (kg/m^3^), *c* defines the specific heat (J/kg/K), and *V* describes the volume (m^3^).

So,

(17) $$\begin{array}{c} \frac{\left(T-T_{b}\right)}{\left(T_{M}-T_{b}\right)}=\exp\left(\frac{-\alpha \times t\times A}{V\times c\times d}\right), \end{array}$$

where *T*_*M*_ describes the initial high temperature. Equation (19) is valid if when *α* × *A* × *t*/*V* × *d* × *c* is *T*-independant,

(18) $$\begin{array}{c} \zeta =\frac{\left(\alpha \times A\right)}{\left(V\times c\times d\right)}. \end{array}$$

So, by considering a constant *ζ*,

(19) $$\begin{array}{c} \frac{T-T_{b}}{T_{M}-T_{b}}=\exp\left(-\zeta t\right). \end{array}$$

So, consequently, by reformulating equation (19),

(20) $$\begin{array}{c} T=\left(T_{M}-T_{b}\right)\times \exp\left(-\gamma t\right)+T_{b}. \end{array}$$

### 6.2. The Algorithm

In Thermal Exchange Optimization, some of the candidates have been assumed as the cooling objects and the other remaining candidates are assumed as the environment; afterward, the reverse operation has been done. In TEO algorithm, like any other metaheuristic algorithm, the candidates are first generated randomly. So, the initial temperature for each object is formulated by the following equation:

(21) $$\begin{array}{c} T_{i}^{0}=T_{\min}+\text{rnd}\times \left(T_{\max}-T_{\min}\right), \end{array}$$

where rnd signifies a random vector limited between 0 and 1, *T*_*i*_^0^ describes the initial solution vector of the *i*^th^object, and*T*_min_ and *T*_max_ represent the minimum and maximum limitations of the candidates.

The randomly generated candidates are then performed to the cost fiction to evaluate their cost value. After that, the position of some best *T* candidate vectors is saved as *Thermal Memory* (TM) to be utilized then for improving the algorithm's efficacy by less computational cost. Afterward, some best TM candidates have been given and the equal numbers of population with the worst values have been eliminated.

**Figure 5:**
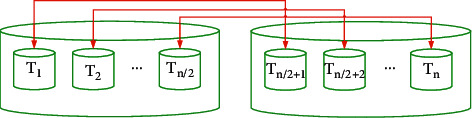
The cooling and heating transfer and environment objects pairs.

The candidates are separated into two equal types that are shown in Figure 5.

For more clarification, *T*_1_ determines the environment object for *T*_*n*/2+1_ cooling object, and, inversely, if any object has lower value than *ζ*, the temperature exchanging is performed slowly. Therefore, *ζ* will be achieved by the following formula:

(22) $$\begin{array}{c} \gamma =\frac{\text{Cos }\left(\text{object}\right)}{\text{Cos }\left(\text{worst object}\right)}. \end{array}$$

Another important term in the algorithm is time, which is associated with the iteration quantity. This parameter is formulated in the following:

(23) $$\begin{array}{c} t=\frac{\text{iteration}}{\text{max. iteration}}. \end{array}$$

To give an exploration term to the algorithm, the environmental temperature changing has been modeled, which is given in the following equation:

(24) $$\begin{array}{c} T_{i}^{e}=\left(1-\left(m_{1}+m_{2}\times \left(1-t\right)\times \text{rand}\right)\right)\times T_{i}^{{'}^{e}}, \end{array}$$

where *m*_1_ and *m*_2_ describe the control variables and *T*_*i*_^'^*e*^^ signifies the object earlier temperature which has been modified to *T*_*i*_^*e*^.

By considering the former models, the new temperature of the objects is updated as follows:

(25) $$\begin{array}{c} T_{i}^{+}=T_{i}^{e}+\left(T_{i}^{\text{old}}-T_{i}^{e}\right)\exp\left(-\zeta t\right). \end{array}$$

The next term is Pr, which is used to indicate whether the cooling objects' element should be altered or not.

The Pr components are compared with *R*(*i*)(*i* = 1,2,…, *n*), which has a randomly distributed value between 0 and 1. If *R*(*i*) < Pr, the *i*^*th*^ candidate will be randomly chosen as 1-dimensional and the value is reformulated by the following:

(26) $$\begin{array}{c} T_{i,j}=T_{j}^{\min}+\text{rnd}\left(T_{j}^{\max}-T_{j}^{\min}\right)\exp\left(-\zeta t\right). \end{array}$$

where *T*_*i*,*j*_ determines the *j*^th^ variable of the *i*^th^ candidate and *T*_*j*_^min^and *T*_*j*_^max^ are the minor and the major limitations of the variable number *j*, respectively. At last, the algorithm is stopped when stopping criteria are checked to terminate the algorithm.

### 6.3. Developed TEO Algorithm

Although TEO algorithm has numerous benefits in providing the best global solution, it also has some drawbacks which could be resolved. The main drawback of the algorithm could be its local minimum results and low convergence in some cases. Numerous improvements were introduced for resolving the exploration of the metaheuristics. In this research, chaotic theory has been utilized as the first improvement. The reason for using chaos in this algorithm is to improve the algorithm's efficiency to resolve the local optimization followed by an advanced speed of convergence. In the considered dTEO algorithm, the modified randk is replaced with the “rnd,” i.e.,

(27) $$\begin{array}{c} {\text{rand}}_{k}=\alpha f_{k}^{2}\sin\left(\pi f_{k}\right),\\ f_{0}\in \left[0,1\right],\;\alpha \in \left(0,4\right], \end{array}$$

where *k* signifies the number of iterations.

The other improvement for the algorithm to give a proper trade-off between exploration and exploitation in the algorithm using the Gaussian mutation mechanism is formulated below [35]:

(28) $$\begin{array}{c} g\left(x\right)=\frac{1}{\sqrt{2\pi \sigma }}\exp\left(-\frac{{\left(x-\mu \right)}^{2}}{2\sigma^{2}}\right), \end{array}$$

where *σ*^2^ signifies the variance of the Gaussian PDF and *μ* determines the Gaussian distribution expectation. This difference is applied to the considered location by renewing the TEO algorithm (*T*_*i*,*j*_^new^) as follows:

(29) $$\begin{array}{c} T_{i,j}^{\text{new}}\;=T_{i,j}\times \left(1+\gamma \times g\left(0,1\right)\right). \end{array}$$

where *γ* describes a decreasing random value between 0 and 1 and *g*(0,1) describes the standard Gaussian distribution.

### 6.4. Algorithm Authentication

In this section, the proposed developed Thermal Exchange Optimization (dTEO) algorithm that is stated in the previous section has been programmed by the MATLAB 2017b environment and then validated based on a 16 GB RAM Core™ i7/4720HQ 1.60 GHz processor. The proposed dTEO algorithm validation is based on applying it to some standard test cases. The results are also compared with some other approaches, that is, Multiverse Optimizer (MVO) [36], Locust Swarm Optimization (LSO) [37], Spotted Hyena Optimizer (SHO) [38], and the original TEO [39], to show the proposed method's efficacy.  Table 2 shows the details of the studied functions.

In Table 3, the parameters settings of all studied algorithms for use in this study are given below.

For determining the performance of the algorithm, they performed on the benchmark functions and two measurement indicators including the minimum value and the standard values are extracted from the results to show the algorithms' accuracy and reliability. It should be noted that, for all algorithms, the population size and the number of iterations are set as 120 and 100, respectively. The simulations have been repeated 20 times to give consistent results.  Table 4 illustrates the simulation results.

As can be seen from Table 4, the suggested dTEO has the least value of the minimum value for the minimization problems. This means that the suggested method provides the best accuracy toward the other state-of-the-art methods for solving the studied functions. Plus, based on the results achieved for the standard deviation, it is definite that the suggested method has the minimum value of that. This shows that using the proposed method has the highest reliability against the comparative techniques in this study. So, we can conclude that using this algorithm in this study is a good idea in terms of accuracy and reliability.

## 7. Optimized ELM Network Based on Algorithm

The classification of the final extracted features is performed based on the proposed optimized ELM network. The main task of the ELM classifier here is to determine the skin cancer dermoscopy image into two groups of normal and malignant melanoma classes.

In this study, activation functions of the network are considered for enhancement. Generally, the significant effect of activation function on the ANNs is too clear, because a network with various activation functions gives good generalization capacities. This study considers a sigmoid activation function as one of the popular functions in this area. The formulation for this function is explained as follows:

(30) $$\begin{array}{c} g\left(X\right)=\frac{1}{1+\exp\left(-\left(\text{as}.x+\text{bs}\right)\right)}. \end{array}$$

**Figure 6:**
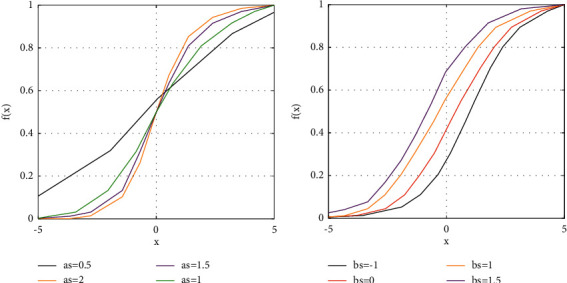
The impact of (a) as and (b) bs on the function shape.

For improving the efficiency of the ELM-based classifier, different sigmoid functions have been utilized in the hidden neurons. In other words, different values are utilized foras and bs.  Figure 6 displays the impact of as and bs on the function shape.

In other words, the problem of finding the suitable parameter of the sigmoid function can be considered as an optimization problem. Here, we used a developed Thermal Exchange Optimization algorithm to refine the issue. However, both “as” and “bs” have almost equal effect on the function shape; we used the “bs” for optimization of the network. Therefore, two optimization policies have been used.

The input weights have been first initialized. Then, the developed Thermal Exchange Optimization algorithm is utilized for optimal parameters selection by assuming the initial weights. The objective function of the developed TEO is based on the square error between the output of the desired value and the network by the following:

(31) $$\begin{array}{c} E=\frac{1}{n}\overset{n}{\underset{i=1}{\sum }}\overset{k}{\underset{j=1}{\sum }}{\left(d_{ji}-y_{ji}\right)}^{2}, \end{array}$$

where *n* and *k* describe the numbers of training samples and the output layers, respectively, and *y*_*ji*_ and *d*_*ji*_ represent the output of the network and the desired value, respectively.

The primary target of the present study is to utilize the mentioned optimized ELM network for diagnosis of the skin cancer. The optimization is using a newly designed metaheuristic, namely, the developed Thermal Exchange Optimization algorithm.

## 8. Results and Discussion

In this paper, a new optimized methodology is used to detect the skin cancer from dermoscopy images.

### 8.1. The Database

**Figure 7:**
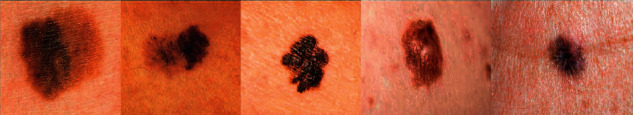
Some examples of the utilized images in the study.

For validating the proposed skin cancer identification system, there are many databases. This analysis uses a well-known dataset, called SIIM-ISIC Melanoma. The images are generated in DICOM format to be retrieved by popular libraries and include both image and metadata. Two formats of TFRecord and JPEG are considered for the dataset. The size of the images is uniform (1024 × 1024). Some examples of the utilized images in the study are displayed in Figure 7.

### 8.2. Results and Discussion

As mentioned before, the present study is authenticated by the ACS database and the results have been verified by some different methodologies. The simulations are performed on a laptop with MATLAB 2018b environment with the following information: 16 GB RAM and Core™ i7-4720HQ-1.60 GHz processor. The pipeline methodology of the method is explained in the following:

1. Start
2. Apply preprocessing
3. Apply noise reduction
4. Apply image contrast enhancement
5. Perform skin cancer segmentation
6. Apply features extraction based on texture features, statistical features, and the geometric features
7. Perform dTEO-based ELM classifier for the final diagnosis
8. Return the results
9. End

In the presented dTEO-based ELM classifier, 80% of data are set to train data, and the remaining 20% are set to test the data. The training step of the network is assumed by 700 iterations. The training process has been iterated 15 times to achieve a confidential result. The validation has been performed using 5 measurement indexes: specificity, sensitivity, accuracy, PPV, and NPV, which are given as follows:

(32) $$\begin{array}{c} \text{accuracy}=\frac{\text{correctly detected cases}}{\text{total cases}},\\ \text{specificity}=\frac{\text{correctly detected healthy skin cases}}{\text{total healthy skin cases }},\\ \text{sensitivity}=\frac{\text{correctly detected skin cancer cases}}{\text{total skin cancer cases }},\\ \text{PPV}=\frac{\text{correctly detected skin cancer cases}}{\text{detected skin cancer cases }},\\ \text{NPV}=\frac{\text{correctly detected healthy skin cases}}{\text{detected healthy skin cases}}. \end{array}$$

The results are compared with 7 other methods, fractal analysis [40], CNN [41], Delaunay Triangulation [42], Side-by-Side method [43], Genetic Algorithm [44], fusion method [45], and SVM [46], to verify the method's performance in different terms.

Table 5 tabulates the performance validation for the suggested technique compared with other methods. The results show the mean value of 30 runs for the methods.

**Figure 8:**
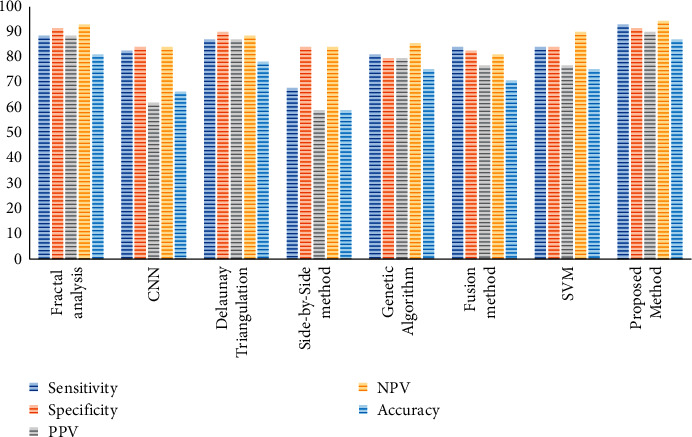
The bar plot of the performance validation for the proposed method compared with other state-of-the-art methods.

As is concluded from Table 5, the suggested method with 95% accuracy provides the uppermost precision among the other compared techniques. Also, with 95% of sensitivity, which is the highest against other methods, the reliability of the proposed method has been proved. This can be proved also for NPV, PPV, and specificity toward the others. The better results of NPV and PPV indicate higher commonness of the condition to determine the probability of a test diagnosing cancer. Also, better results of the specificity and sensitivity for the suggested technique show higher prevalence-independent results for the algorithm. For more clarification, the results of Table 5 are shown in Figure 8.

## 9. Conclusions

In recent years, skin cancer has been recognized as the most dangerous and common type of cancer in humans. There are different types of skin cancer. Melanoma is a common type of skin cancer, where early detection can be helpful in its treatment and can suggestively stop death from this deadly skin cancer. Designing an approach that facilitates the skin cancer detection in the early stages is very useful and valuable. In this study, an optimized pipeline procedure was utilized for the optimal detection of melanoma from dermoscopy images. In the proposed method, after preprocessing of the input dermoscopy images based on noise reduction and contrast enhancement, the region of interest was segmented. Afterward, feature extraction was implemented on the segmented images to extract useful features from them. Finally, an optimized Deep Believe Network (DBN) was utilized to separate images into two classes of healthy and cancerous. The optimization of the DBN was using a new metaheuristic method called the developed Thermal Exchange Optimization algorithm to improve the network efficiency in terms of reliability and accuracy. So, the main contribution of the proposed method is to use a newly developed version of the newly introduced Thermal Exchange Optimization for the diagnosis of malignant melanoma. The main advantage of using this technique is that the results showed that it improved the system's efficiency in both terms of accuracy and precision or, in other words, its reliability during different runs. The performance of the proposed technique was authenticated by comparing it with 7 other methods: fractal analysis, CNN, Delaunay Triangulation, Side-by-Side method, Genetic Algorithm, fusion method, and SVM. Simulation results specified that, based on some various performance indexes, the suggested procedure gave the best results against the compared techniques. The main limitation of the proposed method is that, due to different soft techniques, it needs a lot of time. In the future study, we will work on designing a method to simplify the method from a theoretical methodology to a real-time system for practical applications.

## Figures and Tables

**Algorithm 1:**
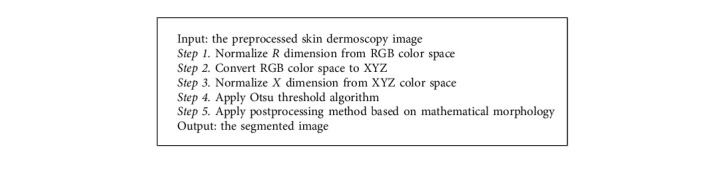
Pseudocode of the proposed image segmentation.

**Table 1:** The utilized features of this study.

Feature name	Formula	Feature name	Formula
Mean	1/MN∑_*i*=1_^*M*^∑_*j*=1_^*N*^*p*(*i*, *j*)	Contrast	∑_*i*=1_^*M*^∑_*j*=1_^*N*^*p*^2^(*i*, *j*)
Variance	1/MN∑_*i*=1_^*M*^∑_*j*=1_^*N*^(*p*(*i*, *j*) − *μ*)	Perimeter	∑_*i*=1_^*M*^∑_*j*=1_^*N*^*B*_*p*_(*i*, *j*)
Standard deviation	$\sqrt{\text{variance}}$	Entropy	−∑_*i*=1_^*M*^∑_*j*=1_^*N*^*p*(*i*, *j*)log *p*(*i*, *j*)
Area	∑_*i*=1_^*M*^∑_*j*=1_^*N*^*p*(*i*, *j*)	Solidity	Area/Convex Area
Rectangularity	Area/*b* × *a*	Correlation	∑_*i*=1_^*M*^∑_*j*=1_^*N*^*p*(*i*, *j*) − *μ*_*r*_*μ*_*c*_/*σ*_*r*_*σ*_*c*_
Irregularity index	4*π* × Area/Perimeter^2^	Invariant moments	*φ*_1_=*η*_20_+*η*_02_
Form factor	Area/*a*^2^		*φ*_2_=(*η*_20_ − *η*_02_)^2^+4*η*_11_^2^
			*φ*_3_=(*η*_30_ − 3*η*_12_)^2^+(3*η*_21_ − *μ*_03_)^2^
Eccentricity	2/*a*(*a*^2^ − *b*^2^)^0.5^	Energy	∑_*i*=1_^*M*^∑_*j*=1_^*N*^*p*^2^(*i*, *j*)
Elongation	$2{\text{Area}}^{0.5}/a\sqrt{\pi }$	Homogeneity	∑_*i*=1_^*M*^∑_*j*=1_^*N*^*p*(*i*, *j*)/1+|*i* − *j*|

**Table 2:** The details of the studied functions.

#	Test function	Minimum value	Boundary
1	*F*_1_=∑_*n*=1_^*N*^*x*_*n*_^2^	0	−*∞* ≤ *x* ≤ *∞*
2	*F*_2_=∑_*n*=1_^*N*−1^(100 × [*x*_*n*+1_ − *x*_*n*_^2^]^2^+[1 − *x*_*n*_]^2^)	0	−*∞* ≤ *x*_*n*_ ≤ *∞*
3	$F_{3}=\sum_{n=1}^{N}\left|x_{n}\right|-10\;\cos\left(\sqrt{\left|10x_{n}\right|}\right)\;$	0	−*∞* ≤ *x*_*n*_ ≤ *∞*
4	*F*_4_=*x* sin(4*x*)+1.1*y* sin (2*y*)	−18.5547	0 ≤ *x*, *y* ≤ 10
5	*F*_5_=[∑_*n*=1_^*N*^*nx*_*n*_^4^]+*N*_*n*_(0,1)	Varies	−*∞* ≤ *x* ≤ *∞*
6	*F*_6_=10*N*+∑_*n*=1_^*N*^[*x*_*n*_^2^ − 10 cos(2*πx*_*n*_)]	0	−*∞* ≤ *x*_*n*_ ≤ *∞*
7	$F_{7}=1+\sum_{n=1}^{N}\frac{x_{n}^{2}}{4000}-\prod_{n=1}^{N}\cos\left(x_{n}\right)$	0	−*∞* ≤ *x*_*n*_ ≤ *∞*
8	$F_{8}=1/2+\sin^{2}\sqrt{x^{2}+y^{2}}-0.5/1+0.1\left(x^{2}+y^{2}\right)$	−0.5231	−*∞* ≤ *x*, *y* ≤ *∞*

**Table 3:** The utilized parameters settings of all studied algorithms.

Algorithm	Parameter	Value	Algorithm	Parameter	Value
LSO [37]	F	0.6	ALO [31]	w	[2, 6]
	L	1		No. of search agents	50
	g	20		Traveling distance rate	[0.6, 1]
SHO [38]	$\overset{⟶}{M}$	[0.5, 1]	MVO [36]	Wormhole existence probability	[0.2, 1]
	$\overset{⟶}{h}$	[5, 0]			

**Table 4:** The comparison results of the studied algorithms implemented on the test functions.

Function	Algorithm						
		LSO [37]	SHO [38]	MVO [36]	ALO [31]	TEO	dTEO
*f*_1_	Min	3.837*e* – 24	2.1201*e* – 28	−2.2800*e* – 27	3.3197*e* – 28	3.4511*e* – 28	8.2193*e* – 31
	Std	2.559*e* – 19	2.4937*e* – 27	4.0865*e* – 28	2.9938*e* – 29	2.0073*e* – 30	2.37928*e* – 32

*f*_2_	Min	7.2874*e* – 5	7.564*e* – 4	4.824*e* – 4	2.56384*e* – 5	4.5463*e* – 6	6.7811*e* – 5
	Std	3.2084*e* – 6	4.18297*e* – 5	2.1167*e* – 5	3.5918*e* – 6	5.0648*e* – 7	2.1253*e* – 5

*f*_3_	Min	−7.2553	−8.15754	−10.00	−7.19376	−10.00	−10.00
	Std	0.35	0.46	0.27	0.15	0.15	0.10

*f*_4_	Min	−5.2846	−18.053	−17.1146	−16.3927	−18.1106	−18.1683
	Std	3.126	1.294	2.391	4.190	1.630	1.01

*f*_5_	Min	11.46*e* – 10	2.597*e* – 16	2.18769*e* – 7	5.0913*e* – 9	2.8096*e* – 23	3.7938*e* – 22
	Std	8.942*e* – 11	4.0973*e* – 17	2.89430*e* – 8	6.5514*e* – 10	2.8897*e* – 25	7.1937*e* – 24

*f*_6_	Min	4.276*e* – 11	2.1953*e* – 12	2.1967*e* – 21	3.1957*e* – 10	2.0054*e* – 18	5.6124*e* – 23
	Std	7.297*e* – 12	1.5374*e* – 14	4.2849*e* – 23	6.8091*e* – 11	2.3169*e* – 19	4.6498*e* – 24

*f*_7_	Min	2.623*e* – 15	3.3733*e* – 10	5.0406*e* – 8	3.7628*e* – 13	3.6781*e* – 9	6.39482*e* – 17
	Std	1.167*e* – 16	4.1967*e* – 12	4.9364*e* – 9	3.1936*e* – 15	4.1945*e* – 10	2.22864*e* – 19

*f*_8_	Min	0.0067	−0.1472	−0.3492	−0.5846	−0.5790	−0.5273
	Std	0.653	0.467	0.385	0.805	0.152	0.100

**Table 5:** The performance validation of the suggested technique compared with other methods.

Method	Performance metric				
	Accuracy	Sensitivity	Specificity	PPV	NPV
Fractal analysis [40]	88.23	91.18	88.23	80.88	92.65
CNN [41]	82.35	83.82	61.76	66.18	83.82
Delaunay Triangulation [42]	86.76	89.70	86.76	77.94	88.23
Side-by-Side method [43]	67.64	83.82	58.82	58.82	83.82
Genetic Algorithm [44]	80.88	79.412	79.41	75.00	85.29
Fusion method [45]	83.82	82.35	76.47	70.58	80.88
SVM [46]	83.82	83.82	76.47	75.00	89.70
Proposed method	92.65	91.18	89.70	86.76	94.12

## Data Availability

The data are available at https://www.kaggle.com/c/siim-isic-melanoma-classification.
